# A promising self-nanoemulsifying adjuvant with plant-derived saponin D boosts immune response and exerts an anti-tumor effect

**DOI:** 10.3389/fimmu.2023.1154836

**Published:** 2023-06-21

**Authors:** Xing Luo, Zhen Song, Xiaogqiang Zeng, Yan Ye, Hailin Zheng, Dingyi Cai, Qingpeng Yuan, Haibo Li, Yanan Tong, Dongshui Lu, Yuheng Liu, Hao Zeng, Yun Yang, Hongwu Sun, Quanming Zou

**Affiliations:** ^1^ National Engineering Research Center of Immunological Products, Department of Microbiology and Biochemical Pharmacy, College of Pharmacy, Third Military Medical University, Chongqing, China; ^2^ Department of Clinical Laboratory, The 954 Army Hospital, Shannan, Tibet, China; ^3^ Department of Nuclear Medicine, General Hospital of Northern Theater Command, Shenyang, China

**Keywords:** plant adjuvant, saponin D, self nanoemulsifying adjuvant, immune response, tumor effect

## Abstract

**Objectives:**

The low immunogenicity of tumor antigens and unacceptable toxicity of adjuvants has hindered the application and development of tumor vaccines. Hence, we designed a novel anti-tumor vaccine composed of a plant-derived immunostimulant molecular nanoadjuvant (a self-nanoemulsifying system, SND) and the antigen OVA, to reinvigorate the immune response and inhibit tumor progression.

**Methods:**

In this study, this novel nanoadjuvant with Saponin D (SND) was designed and prepared by low-energy emulsification methods. Several important characteristics of the SND, including morphology, size, polymer dispersity index (PDI), zeta potential, and stability, were estimated, and the cytotoxicity of the SND was evaluated by MTT assay. Additionally, the immune response in terms of antibody titer levels and cellular immunity were evaluated *in vivo* after immunization with the vaccine, and the preventative and therapeutic effects of this novel vaccine against tumors were estimated. Finally, the antigen release profile was determined by IVIS imaging and by *in vivo* assay.

**Results:**

This SND nanoadjuvant had good characteristics including the average particle size of 26.35 ± 0.225 nm, narrow distribution of 0.221 ± 1.76, and stability zeta potential of -12.9 ± 0.83 mV. And also, it had good stability (size, PDI, zeta potential, antigen stability) and low toxicity *in vitro* and *in vivo*, and delayed antigen release *in vivo*. The humoral immune response (IgG, IgG1, IgG2a, and IgG2b) and cellular immune level (cytokines of splenocytes including IFN-γ, IL-4, IL-1β andIL-17A) were both improved greatly after injected immunization at 0, 14, 28 days with the novel nanoadjuvant and antigen OVA. Importantly, this novel nanoadjuvant combined with OVA might lead to the induction of the prevent and treatment efficacy in the E.G7-OVA tumor-bearing mice.

**Conclusions:**

These results suggested that this novel nanoadjuvant encapsulated natural plant immunostimulant molecular OPD could be a good candidate of tumor vaccine adjuvant for reinvigorating the immune response and powerfully inhibiting tumor growth effect.

## Introduction

Tumor vaccines have become a powerful immunotherapeutic strategy owing to their specific preventive and therapeutic performance in directing the immune system to eradicate tumor cells (1–5). Tumor-specific immune responses are activated by tumor vaccines that deliver tumor antigens to antigen-presenting cells (APCs) in the lymph nodes, and potent anti-tumor immunity and long-term immune memory are induced to inhibit tumor progression (4–6). Over the past decade, several tumor vaccines have been successfully used clinically and have shown good efficacy. Many immunotherapy candidates are currently being tested in preclinical or clinical trials (3, 7–11). However, numerous trials have shown minimal efficacy due to low immunogenicity of the tumor antigen or unacceptable toxicity of the adjuvants (3).

Saponins have shown potential for use as vaccine adjuvants due to their ability to activate the immune system (12, 13). In a recent study, a recombinant SARS-CoV-2 vaccine candidate based on saponin adjuvant was found to be capable of inducing an effective Th1/Th2 immune response (14). A saponin adjuvant, QS-21, has also been used in a therapeutic breast tumor vaccine and has been found to generate strong anti-tumor activity and immune responses (15). Ophiopogonin D (OPD), a vital bio-active steroidal glycoside that comes from the root of *O. japonicas*, has exhibited a broad range of pharmacological properties, including anti−inflammatory effects, anti-tussive effects, and inhibition of venous thrombosis (16, 17). It has been considered as a adjuvant candidate because of its monodesmoside structure (18). However, its poor solubility in water, undesirable level of toxicity, and hemolytic effects severely hinder its use in human vaccination (15).

With the development of nanotechnology and materials science, nanobiomaterials have received extensive attention and have shown great potential in improving adjuvant effects (19). Previously reported data have suggested that nano-scale drug delivery systems can increase antigen uptake by dendritic cells (DCs), thereby promoting the initiation of an antigen-specific immune response (19, 20). Moreover, the design of tumor vaccines using nanomaterials as the drug delivery tool has been confirmed as an effective strategy to induce anti-tumor immunity (21–23). In a representative study, it was found that a self-assembled polymer with low agonist density with a diameter range of 10–20 nm can induce higher cytokine production in the lymph node than observed in a control group (24), indicating that nano-scale particles can effectively enhance the T cell immune response. Additionally, studies have proven that nano-scale adjuvants used in tumor vaccines can effectively reduce their toxicity as well as enhancing their anti-tumor efficacy, especially in relation to T cell immunity (25, 26). Therefore, nano-scale adjuvants are superior choices for the design of tumor vaccines. In our previous study, we designed a novel nanoemulsion adjuvant (nanoemulsion-encapsulated OPD, NOD), which exhibited a robust adjuvant effect and significantly improved vaccine efficacy against MRSA (20). However, it is a key question that this nature plant immunomodulators adjuvant of OPD with poor solubility whether can induce the cellular immune response especially tumor vaccine.

To address the issue of poor solubility in water and to evaluate the adjuvant effect of OPD, in this study, a plant-derived immunostimulant molecular nanoadjuvant, self-nanoemulsifying with OPD (a self-nanoemulsifying drug, or SND), was designed and prepared by low-energy emulsification methods. A novel anti-tumor vaccine, consisting of the SND and the model antigen OVA, was also designed to reinvigorate the immune response and inhibit tumor progression. Several important characteristics of the SND, including morphology, size, polymer dispersity index (PDI), zeta potential, and stability, were estimated, and the cytotoxicity of the SND was evaluated by MTT assay. Additionally, the immune response in terms of antibody titer levels and cellular immunity were evaluated in mice after immunization with the vaccine, and the preventative and therapeutic effects of this novel vaccine against tumors were estimated. Finally, the antigen release profile was determined by IVIS imaging and by *in vivo* assay.

## Materials and methods

### Cells, animals, and ethics statement

Both types of mouse lymphocytes used in the experiment, namely DC2.4 cells and E.G7-OVA cells, were purchased from the American Type Culture Collection (ATCC). BALB/c mice and C57BL/6 mice (SPF, female, 6–8 weeks of age) were purchased from Beijing HFK Bioscience and Hunan SJA Laboratory Animal Co. (China), respectively. The animal experimental protocols were approved by the Laboratory Animal Welfare and Ethics Committee of the Third Military Medical University and performed according to the Guide for the Care and Use of Laboratory Animals (27); professional technicians, skilled operation of equipment, and suitable experimental conditions for the animals were ensured in order to minimize pain experienced by the mice.

### Reagents

Ophiopogonin D (catalog: 945619-74-9) was purchased from Chengdu Purui Technology Co. Ltd (China). Mouse IL-1β enzyme-linked immunosorbent assay (ELISA) kits (catalog: 1210122), mouse IFN-γ ELISA kits (catalog: 1210002), mouse IL-4 ELISA kits (catalog: 1210402), mouse IL-17A ELISA kits (catalog: 1211702) and mouse IFN-γ precoated enzyme-linked immunospot assay (ELISpot) kits (catalog: DKW22-2000-096) were purchased from Dakewe Biotech (China). Mouse IL-17A ELISpotPLUS kits (catalog: 3521-4HPW-2) were purchased from Ebiosciences (USA). Fetal bovine serum (FBS) (catalog: 10099141C) was purchased from GIBCO (USA). RPMI 1640 medium (catalog: SH30809.01) was purchased from Hyclone (Life Technology, USA). Bovine serum albumin (BSA) (catalog: A8020-100g) and phalloidin (catalog: CA1620) were purchased from Solarbio (China). Glutamax (catalog: 35050061), HEPES (catalog: 15630106), penicillin–streptomycin solution (catalog: 15070063), sodium pyruvate (100 mM) (catalog: 11360070), and β-mercaptoethanol (catalog: 21985023) were purchased from Invitrogen (USA).

### Preparation of the self-nanoemulsifying system of saponin D

In accordance with our previously reported method (20, 28, 29), the novel self-nanoemulsifying system of saponin D was designed and developed with low-energy emulsification. Briefly, surfactant Tween 80, glycerol, and oil-phase caprylic/capric triglyceride (GTCC, Beijing Fengli Pharmaceutical Co. Ltd., Beijing, China) with a mass ratio of 10:2:3 were weighed precisely in a small 50 mL beaker and mixed thoroughly. Subsequently, OPD and deionized water (w/w = 1:6) were added dropwise at a constant speed and the mixture was stirred at a constant speed in a clockwise direction to obtain SND. The resulting novel self-nanoemulsifying system of saponin D, containing 1mg/mL, was of a clear, transparent, and fluid form. A blank self-nanoemulsifying (BSN) formulation was prepared *via* the same methods, with the OPD entirely replaced with deionized water.

### Characterization of the self-nanoemulsifying system of saponin D

The morphology of the SND was observed using transmission electron microscopy (TEM) and scanning electron microscopy (SEM). TEM images were acquired using a Tecnai 10 (Philips, Holland). The SND (1 mg/mL) was diluted at 1:200 with deionized water and adsorbed onto a copper grid for 2 h, then stained with 2% phosphotungstic acid for 30 min, dried naturally, and observed on the machine. SEM images were acquired using an AMRAY 1000B (AmRay Incorporated, Bedford, MA, USA). The silicon wafers were soaked in 75% ethanol for 30 min in advance. The SND was diluted with deionized water at 1:200 and then dropped on the dry wafer surface and left for 2 h. The samples were sprayed with gold, dried, and then loaded on the machine for observation. The surface structure of the SND was determined by atomic force microscopy (AFM) using an IPC-208B (Chongqing University, Chongqing city, China). The diameter and surface charge of the SND were measured using a Zetasizer Nano ZS (Malvern Instruments Ltd, UK).

### Physical state and drug interaction of the nanoadjuvant

Differential scanning calorimetry (DSC) and thermogravimetric analysis (TGA) of the suspension (OPD) and the self-nanoemulsifying system of saponin D (an SND) were conducted using the TA Instruments Q600 system (New Castle, USA) at a 10°C/min heating rate from room temperature up to 300°C in a nitrogen atmosphere (30). Fourier-transform infrared (FTIR) spectroscopy absorption data were obtained using a Lambda 950 spectrometer (Perkin Elmer, Boston, USA) with a resolution of 4 cm^−1^ for 64 scans in the spectral range of 400–4000 cm^−1^.

### Stability of the nanoadjuvant and influence on OVA protein

SND (1 mg/mL) was diluted at 1:100 with deionized water, and its size, zeta potential, and PDI were measured after it had been kept at room temperature for 0, 7, 14, 21, and 28 days. To test whether OPD, BSN, and SND would disrupt the protein structure of the antigen, the three samples were physically adsorbed to the antigen (w/w = 3:10) at 4°C for 2 h using the model protein OVA as the antigen. A naked antigen group was also prepared by replacing the adjuvant with physiological saline. Four groups of samples were diluted at 1:20 in normal saline and heated with 5 × protein loading buffer (v/v = 4:1) at 100°C for 6 min before loading on 10% SDS-polyacrylamide gels, and electrophoresis was subsequently run at 60 V. The gels containing samples were stained with instant blue for 2 h, destained overnight on a shaker, and images were taken on a gel imager (Bio-Rad, USA).

### Cytotoxicity of the self-nanoemulsifying system of saponin D

DC2.4 cells line were grown in completed 1640 medium (10% FBS, 1% penicillin–streptomycin solution) at 37°C in 5% CO_2_. When cells were in the logarithmic growth phase, they were placed in 96-well plates (1×10^4^/well) and marginal wells were filled with PBS to reduce the evaporation rate. Samples at different concentration gradients of OPD and SND (diluted 11-fold downwards from 0.5 mg/mL with a 2-fold gradient), with 5 replicate wells per group, were added at 100 μL/well after 24 h of culture. At 24 h after treatment, 20 μL of 5 mg/mL MTT solution was added and the samples were incubated for 4 h. After the supernatant had been discarded, 150 μL/well of dimethylsulfoxide (DMSO) was added and the samples were shaken (100 rpm, 10 min) to dissolve the crystals completely. The absorbance of the samples was measured at OD_490_ nm *via* enzyme-linked immunosorbent assay (Thermo Fisher Scientific, USA).

### Antibody level of the nanoadjuvant combination with OVA

BALB/c mice were divided randomly into 5 groups (n=7) and injected intramuscularly with PBS, OVA, OVA/BSN, OVA/OPD, or OVA/SND at 0 days, 14 days, and 28 days, with 20 μg/mouse and 50 μg/mice for the antigen and adjuvant doses, respectively. Mouse serum samples were collected at 7 days, 21 days, and 35 days for detection of antibody titers of IgG. Additionally, IgG1, IgG2a and IgG2b, IgG1/IgG2a were determined at 35 days. Serum samples were diluted with antibody diluent starting at 1:400 and diluted 11-fold back to 1:1. These pre-diluted samples were added to plates at 100 μL/well after coating with OVA (10 μg/mL) solution and incubated statically at 37°C for 1 h. Plates were then washed 3 times with 300 μL/well PBST and patted dry. Subsequently, 100 μL/well was added along with pre-diluted goat anti-mouse IgG (v/v = 1:10000), IgG1 (v/v = 1:5000), IgG2a (v/v = 1:5000), and IgG2b (v/v = 1:5000) antibody solutions and kept statically at 37°C for 40 min. Plates were then washed 3 times with PBST at 300 μL/well and patted dry. Finally, 100μL/well of TMB chromogen solution was added, plates were incubated at 37°C in the dark for 8–12 min, and 2 M H_2_SO_4_ at 50 μL/well was terminated. The absorbance of these samples at OD_450_ nm was measured *via* enzyme-linked immunosorbent assay.

### Cytokine level for the nanoadjuvant combination with OVA

Seven days after the last immunization, all mice spleens were taken for grinding. Splenocyte suspensions were diluted in 1640 complete medium containing 1% penicillin–streptomycin solution, 10% FBS, 1% non-essential amino acids, 1% sodium pyruvate, 2 mM glutamax, 0.05 mM 2-mercaptoethanol, and 10 mM HEPES, and seeded at 1 × 10^6^/well in 24-well plates with or without stimulation with OVA_257-264_ (10 μg/mL). Splenocyte supernatant was obtained for the determination of cytokines IL-1β, IFN-γ, IL-4, and IL-17A after 3 days of culture at 37°C, 5% CO_2_.

### Frequency of IFN-γ and IL-17A producing cells

The number of cells induced to produce IFN-γ and IL-17A was counted primarily using mouse precoated ELISpot kits. The diluted cell suspension described above was seeded into ELISpot plates at 1 × 10^6^/well and incubated with 10 μg OVA_257-264_ peptide for 72 h at 37°C, 5% CO_2_. Plates were washed 5 times with PBS at 200 μL/well. Subsequently, 100 μL/well of IFN-γ and IL-17A detection antibodies diluted 1:1000 was added and the plates were left in place for 2 h in a dark room. Next, streptavidin–horseradish peroxidase (1:1000) was added at 100 μL/well and the plates were incubated for 1 h in a dark room after being washed 5 times with PBS. Finally, 100 μL/well was developed by adding TMB for about 15 min until significant spots appeared, and samples were then rinsed with deionized water. Spot counts were performed using a QC-026-MROI ELISpot plate reader (AID, Germany).

### Th1/Th2/Th17 levels for the nanoadjuvant with OVA

Mice serum samples were collected 7 days after the last immunization and cytokine levels were measured. Mouse cytokine grp I panel 23-plex liquid-phase suspension microarray cytokine levels, including Th2/Th17 immune response (IL-4, IL-5, IL-6,IL-9, IL-10 and IL-17A) and Th1 immune response (IL-1α, IL-1β, IL-2, IL-3, IL-12p40, IL-12p70, IL-13, eotaxin, G-CSF, GM-CSF, IFN-γ, CXCR1, MCP, MIP-1α, MIP-1β, RANTES and TNF-α), were obtained using a Luminex 200™ system (Luminex Corporation, Netherlands) with a Bio-Plex Handheld Magnetic Washer (Bio Rad, USA). All data were processed using the Bio-Plex Manager software (Ver. 8.1, Bio Rad, USA).

### Preventative effects against tumor

To investigate preventative effects against tumor, sixty C57BL/6 mice were divided randomly into 5 groups and injections with PBS, OVA, OVA/SND, OVA/OPD, or OVA/BSN were administered at 0, 7, and 14 days, with doses for both antigen and adjuvant of 50 μg/mouse. On day 21, lymphocyte E.G7-OVA cells were injected into the right shoulder of each mouse. To enable better evaluation of preventative immuno-protection, no mouse deaths should occur throughout the experiment except among the survival analysis group. Therefore, the tumor-catching dose for mice was mapped and a high-dose group (survival rate analysis: 4 × 10^5^/mouse in 100 μL of blank 1640 medium) with a clear survival trend and a low-dose group (tumor pathology analysis: 1.5 × 10^5^/mouse in 100 μL of blank 1640 medium) with a significant difference in tumor volume were designed. Body weights and tumor volumes of all mice were measured every 3 days. Tumor volume for each mouse was calculated as (L x W^2^)/2, where L represents the length and W the width. Mice were considered to be dead once the tumor volume exceeded 2000 mm^3^ (31). The mice in the survival rate analysis group were observed for 60 days, and the mice in the tumor pathology analysis group were observed for 18 days. On day 18, the tumor nodules were removed from surviving mice in the pathology analysis group. Tumor specimens were treated with 10% formalin solution and embedded with dehydrated solution. Subsequently, tumor necrosis was assessed after staining of thin sections with hematoxylin and eosin (HE).

### Therapeutic effects against tumor

To study therapeutic effects against tumor, lymphocyte E.G7-OVA cells were injected into mice in the back of the right shoulder on day 0. To enable better evaluation of therapeutic immuno-protection, no mouse deaths should occur throughout the experiment except among the survival analysis group. Therefore, the tumor-catching dose for mice was mapped and a high-dose group (survival rate analysis: 4 × 10^5^/mouse in 100 μL of blank 1640 medium) with a clear survival trend and a low-dose group (tumor pathology analysis: 1.5 × 10^5^/mouse in 100 μL of blank 1640 medium) with a significant difference in tumor volume were designed. Seventy C57BL/6 mice were divided randomly into 5 groups and injected on days 3, 10, and 17 with PBS, OVA, OVA/BSN, OVA/OPD, OVA/SND, where antigen and adjuvant doses were both 50 μg/mouse. Body weights and tumor volumes of all mice were measured every 3 days. Tumor volume of each mouse was calculated as (L x W^2^)/2, where L represents the length and W the width. Mice were considered to be dead once the tumor volume exceeded 2000 mm^3^ (31). The mice in the survival rate analysis group were observed for 21 days, and the mice in the tumor pathology analysis group were observed for 18 days. On day 18, the tumor nodules were removed from surviving mice in the pathology analysis group. Tumor specimens were treated with 10% formalin solution and embedded with dehydrated solution. Subsequently, tumor necrosis was assessed after staining of thin sections with hematoxylin and eosin.

### IVIS measurement of antigen persistence of the nanoadjuvant

Cy5.0-OVA was used as vaccine antigen for IVIS (*in vivo* imaging system) observation. Nine BALB/c mice were injected subcutaneously in the neck with Cy5.0-OVA, Cy5.0-OVA/OPD, and Cy5.0-OVA/SND at antigen and adjuvant doses of 5 μg/mouse and 50 μg/mouse, respectively. Cy5-derived fluorescence intensity at the injection site was measured at multiple time points (0, 1, 2, 8, 12, 24, 48, 96, 192, and 384 h) using an IVIS Lumina LT system (Caliper Life Science Limited Company).

### Statistical analysis

One-way ANOVA and Tukey’s multiple comparison tests were used to test for differences between multiple groups. The log-rank (Mantel–Cox) test was conducted to compare the survival rate in each group of mice. All data are presented in the form average ± SD (standard deviation); data were processed using the software GraphPad Prism 8.0. Significant differences are indicated as follows: ****P < 0.0001; ***P < 0.001; **P < 0.01; *P < 0.05.

## Results

### Preparation of the self-nanoemulsifying system of saponin D

**Figure 1:**
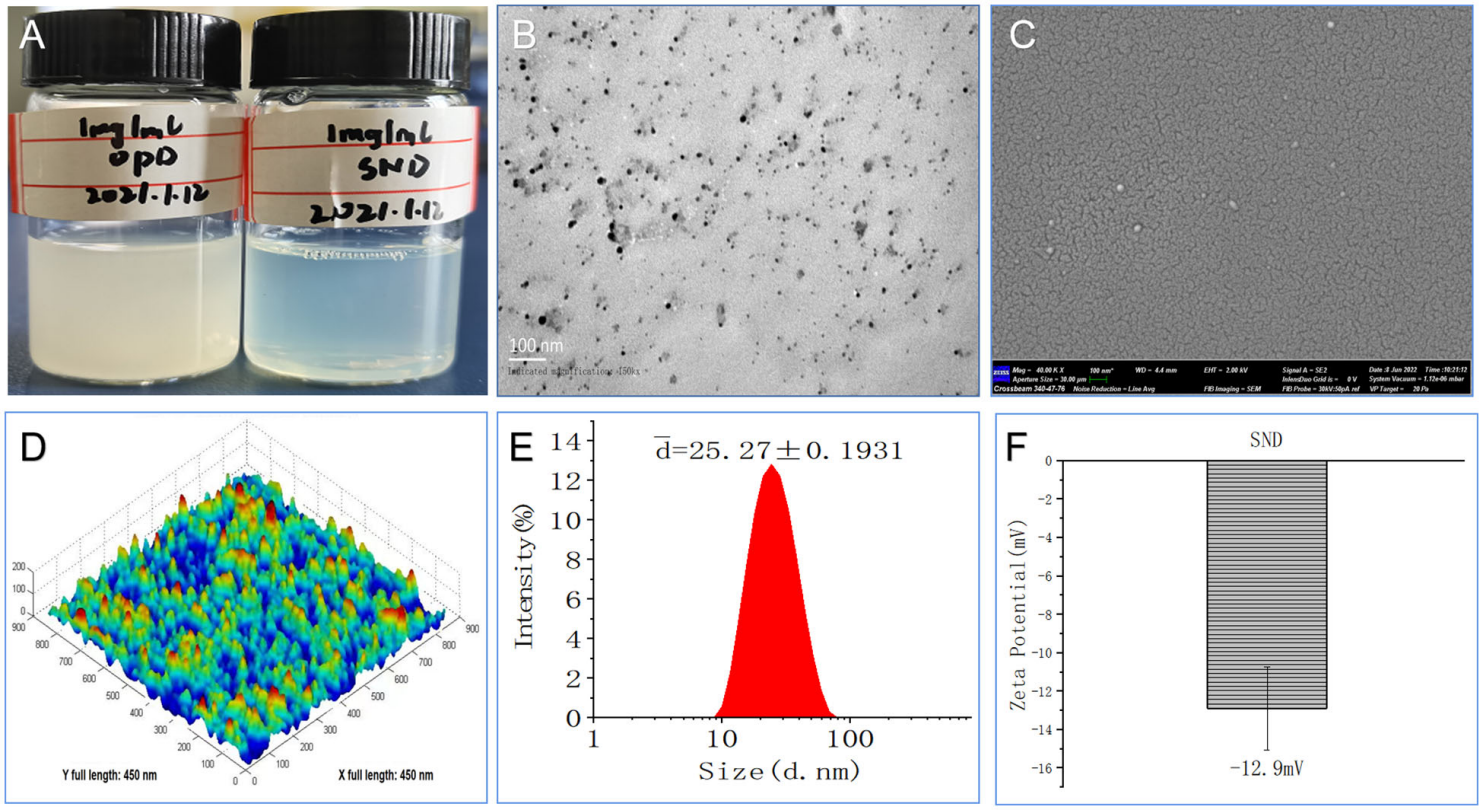
Morphological and physicochemical characteristics of this nanoadjvuant. **(A)** Appearance of 1 mg/ml with OPD and SND. **(B)** Transmission electron micrograph. **(C)** Scanning electron micrograph. **(D)** Atomic force microscopy micrograph. **(E)** Size diameter and distribution. **(F)** Zeta potential and distribution.

The novel OPD-loaded self-nanoemulsifying system (an SND), composed of tween 80, glycol, and GTCC, was designed and prepared using the low-energy emulsification method. Regarding visual appearance, the OPD suspension with 1 mg/mL was a suspended and turbid solution, while the SND formulated with the novel self-nanoemulsifying system with 1 mg/mL was a transparent and clear liquid (**Figure 1A**). This result indicates that the solubility of the SND formulation was significantly improved (OPD< 20 μg/mL, less than 50-fold).

### Characterization of the self-nanoemulsifying system of saponin D

As shown in **Figure 1B**, some dark droplets appeared in the SND under exposure to a bright environment, and a “positive” image was observed using a transmission electron microscope. The SEM images also showed that the coated particles of these samples were spherical and evenly distributed, with a rough surface, depression, and wave crest (**Figure 1C**). Furthermore, the ultrastructure of the SND, as observed in the AFM images, indicated that most of the droplets were spherical with good dispersion (**Figure 1D**). Assessment of a narrow and good distribution was based on the mean size (25.27 ± 0.1931 nm) and PDI value (0.274 ± 0.005, PDI < 0.3), as shown in **Figure 1E**. The average zeta potential of this nanoadjuvant was -12.9 mV (**Figure 1F**). These results further confirm that the self-nanoemulsifying system loaded with OPD had high-quality characteristics.

### Physical state and drug interaction of the nanoadjuvant

**Figure 2:**
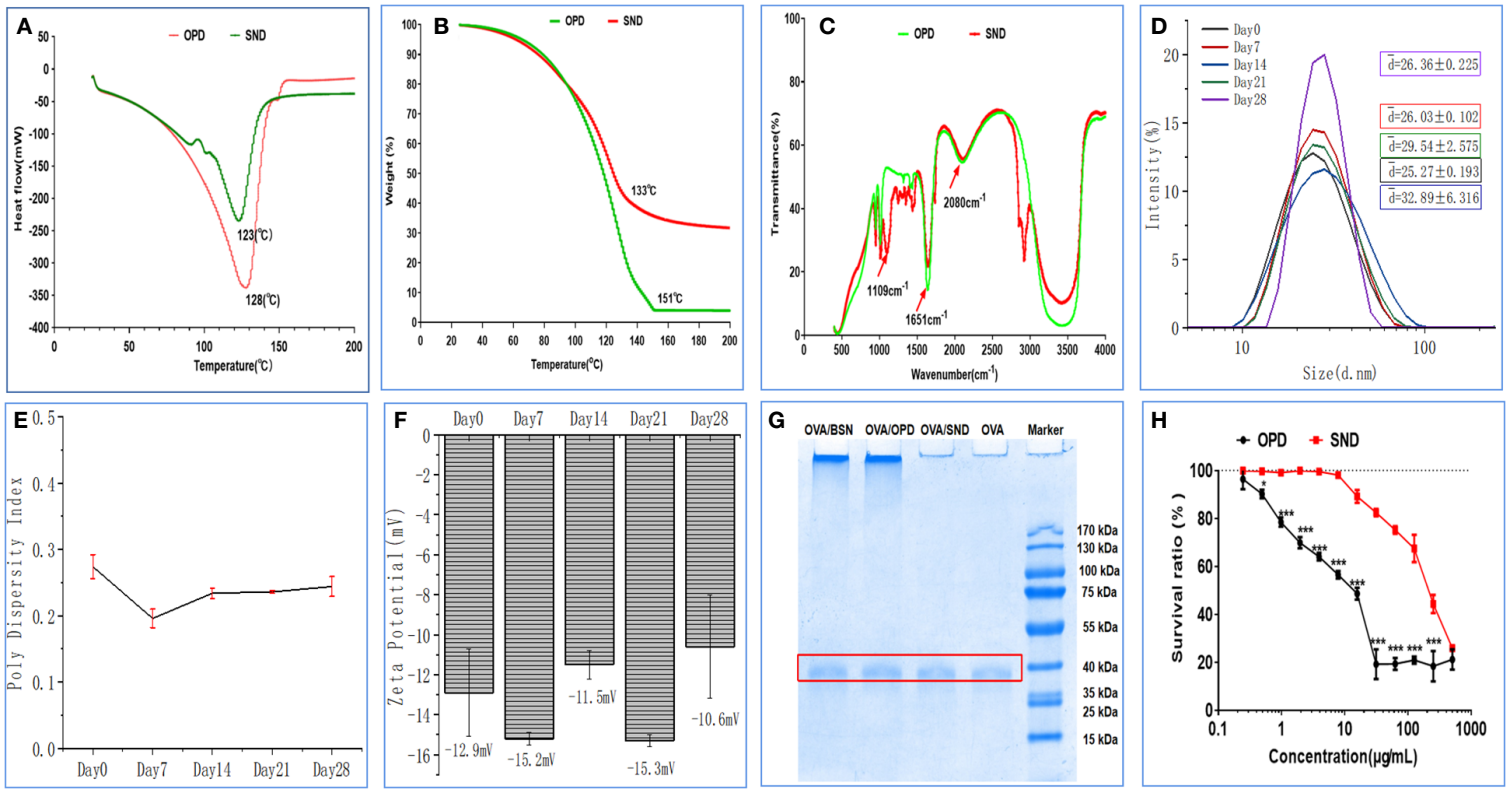
Stability and cytotoxicity of the nanoadjuvant. **(A)** Differential scanning calorimetry. **(B)** Thermogravimetric analysis. **(C)** FTIR spectrum. **(D–F)** Changes in size, PDI, and zeta potential of the adjuvant when stored at room temperature. **(G)** OVA protien structral damage caused by the nanoadjuvant. **(H)** DC2.4 cytotoxicity of the nanoadjuvant. The results are shown in the form mean ± SD(n=3), *P < 0.05; ***P < 0.001.

DSC curves for OPD and SND are shown in **Figure 2A**. We found that a characteristic and significant peak value was observed for OPD at 128 °C, but this peak did not occur in the case of the SND formulation. In addition, a peak of 123°C was observed for the SND formulation, indicating that successful loading of OPD caused a shift in the peak. As shown in **Figure 2B**, weight loss for both OPD and SND began at approximately 30°C and a similar pattern of weight loss was observed in both cases, with both curves showing a step. The peak values for OPD and SND (representing the highest rate of weight loss) occurred at around 151°C and 133°C, respectively. **Figure 2C** shows the FTIR spectra for OPD and SND. OPD exhibited two main characteristic peaks at 1651 cm^-1^ and 2080 cm^-1^, corresponding to C=O stretching and C-H stretching, respectively, and these two characteristic peaks were also present in the SND. In addition, the FTIR spectrum of the SND indicated that the dominant peak observed at 1109 cm^-1^ was attributable to O-H stretching, with no corresponding change found in OPD. These results confirmed that the loading of OPD into the self-nanoemulsifying system was successful.

### Stability of physical characteristics of the nanoadjuvant

The results regarding important physical properties of the SND (particle size, PDI, and zeta potential) are shown in **Figures 2D–F**, as measured at room temperature on days 0, 7, 14, 21, and 28. The particle size of this nanoadjvuant was in the range of 25.27–32.89 nm, as shown in **Figure 2H**. PDI values indicated very little fluctuation in the range of 0.27–0.17 (**Figure 2E**). Finally, as shown in **Figure 2F**, zeta potential of the SND varied from -15.3 mV to -10.6 mV; this indicated high stability. The results showed that there was no significant change after 28 days of storage at room temperature (all *P*s > 0.05).

### Damage to the OVA protein structure caused by the nanoadjuvant

The results on damage to the structure of the OVA protein are shown in **Figure 2G**. OVA was adsorbed with OPD, BSN, SND, and normal saline to determine whether the ovaries were damaged or not. SDS-PAGE produced clear and bright bands between 35 kDa and 40 kDa, and there was no significant difference between naked OVA. These results show that the self-nanoemulsifying system with OPD caused no damage to the OVA protein antigen.

### Cytotoxicity of the self-nanoemulsifying system of saponin D

We performed an MTT assay in DC2.4 cells to detect the cytotoxic effects of OPD and SND, as shown in **Figure 2H**. Under concentrations of 0.24 and 400 μg/mL, SND exhibited a higher survival ratio and lower cytotoxicity, whereas at concentrations of 0.24 and 3.9 μg/mL, OPD exhibited no significant cytotoxicity. These results indicate that encapsulation of OPD in the self-nanoemulsifying system could significantly reduce the toxicity to DC2.4 cells, suggesting that the SND is very safe (survival ratio >90%) at 24 μg/mL.

### Antibody levels for the nanoadjuvant in combination with OVA

**Figure 3:**
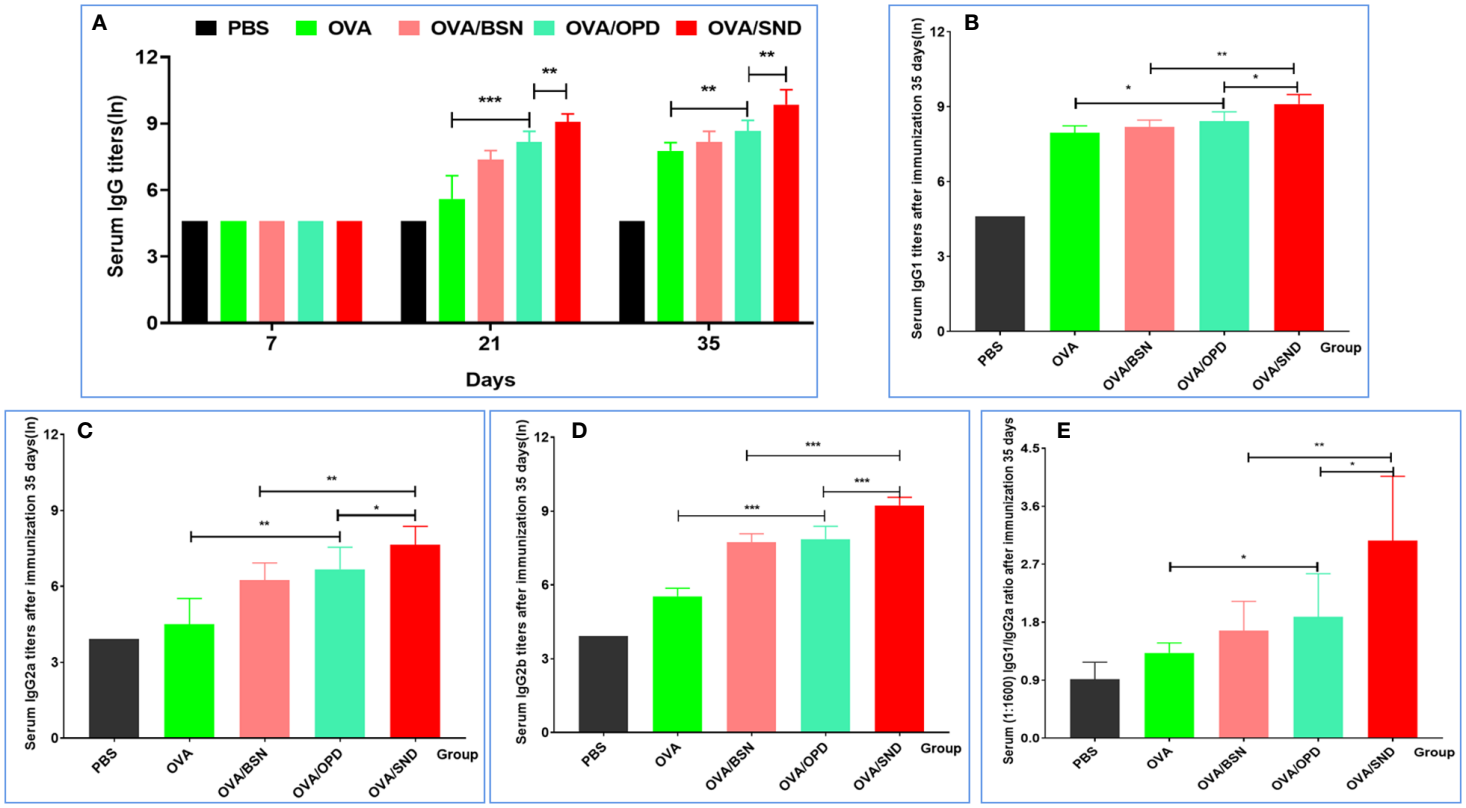
Antibody response level of the nanoadjuvant in combination with OVA. **(A)** Serum samples (n=7) were collected on days 7, 21, and 35. IgG responses were detected by ELISA. **(B–D)** Serum samples (n=7) were collected after immunation 35 days. IgG1, IgG2a, and IgG2b responses were measured by ELISA. Antibody response is expressed as mean ln(titers) ± SD. **(E)** The IgG2a/IgG1 ratio (diluted at 1:1600 with serum OD) was calculated. *P<0.05; **P<0.01; ***P<0.001.

Immunoglobulin G (IgG), as the primary immunoglobulin, is an important indicator for determining the level of immune response. IgG1 and IgG2 are the two main subtypes of IgG1/IgG2a stimulates the Th1 predisposition of the immune response. We used the ELISA method to measure specific IgG antibody titers in serum on days 7, 21, and 35 (**Figure 3A**). We found that antigen-induced IgG titers were significantly higher for the SND in combination with OVA than those induced by OVA/OPD, OVA/BSN, or OVA antigens alone (*P*<0.001) at 21 days after immunization. In addition, serum IgG, IgG1, IgG2a, and IgG2b titers at day 35 were the highest in the OVA/SND group, and significantly higher than in other controls (*P<*0.01) (**Figures 3A–D**). Additionally, we found that the IgG1/IgG2a ratio was significantly higher in this group than in all control groups (*P*<0.05) (**Figure 3E**), suggesting that OVA/SND could enhance the Th1 cellular immune response.

### Cytokine levels for the nanoadjuvant in combination with OVA

**Figure 4:**
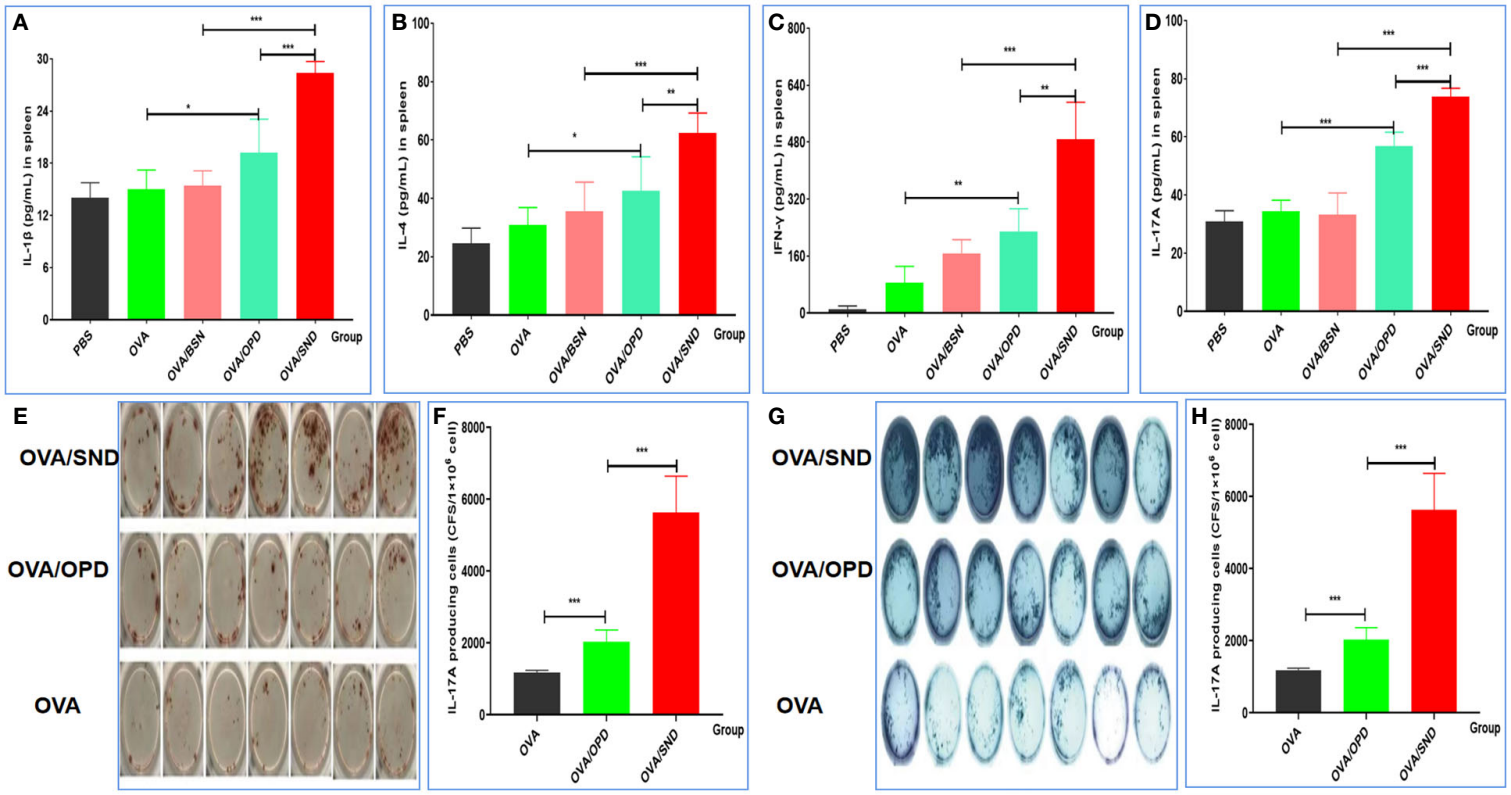
Cellular immune response to the nanoadjuvant in combination with OVA. Splenocytes of immunized mice (n = 7) were stimulated with antigen for 3days. IL-1β **(A)**, IL-4 **(B)**, IFN-γ **(C)**, IL-17A **(D)** were detected by ELISA. **(E, F)** ELISpot figure and analysis of IFN-γ spot-forming cells among splenocytes. **(G, H)** ELISpot figure and analysis of IL-17A spot-forming cells among splenocytes. The results are shown in the form mean± SD. *P < 0.05; **P < 0.01; ***P < 0.001.

To further verify the ability of the SND to generate a robust immune response *in vivo*, we continued to evaluate cytokine release from splenocytes of immunized mice after stimulation with the SND combined with the OVA antigen. **Figures 4A–F** show the results for the four types of cytokines detected, namely IL-1β (pro-inflammatory cytokine, **Figure 4A**), IL-4 (Th2-biased cytokine, **Figure 4B**), and IFN-γ (Th1-biased cytokine, **Figure 4C**) and Th17 (Th17-associated cytokine, **Figure 4D**). Compared with OVA/OPD, OVA/SND was found to be capable of stimulating the level of IL-1β, IL-4, IFN-γ and Th17; in particular, there was a significant difference between the OVA/SND group and the OVA/BSN group (all *P*s *<* 0.001).

### The frequency of INF-γ-and IL-17A producing cells

Compared with the antigen alone, OVA/SND increased the levels of IFN-γ and IL-17A ELISpot counts in splenic lymphocytes by 9.74 and 5.24 times, and there were significant differences between OVA/SND and OVA/OPD (*P*<0.001, *P*<0.001) (**Figures 4E–H**). These results further indicated that the nanoemulsifying nanoadjvuant of OPD enhanced Th1 and Th17 cytokine profiles, respectively. In summary, antigens formulated with the SND induced strong Th1 and Th17 cell responses, mainly in the form of Th1-/Th17-tilted immune responses.

### Th1/Th2/Th17 cellular immune response of the nanoadjuvant in combination with OVA

**Figure 5:**
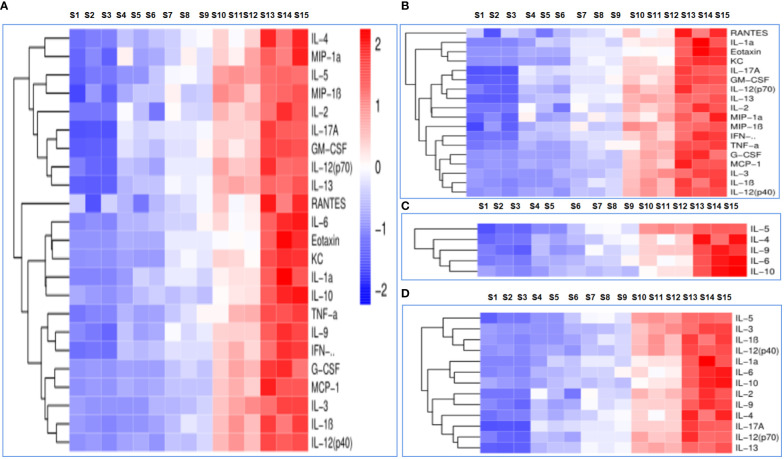
The immune response to the nanoadjuvant in combination with OVA. **(A)** Each cytokine. **(B)** Th1 immune response. **(C)** Th2 immune response. **(D)** Inflammatory cytokines. S1–S3: PBS; S4–S6: OVA; S7–S9: OVA/OPD; S10–S12: OVA/SND; S13–S15: OVA/BSN.

Cytokine levels were assessed in order to determine immune responses; the serum cytokine profiles of mice administered PBS, OVA, OVA/BSN, OVA/OPD, and OVA/SND are shown in **Figure 5A**. In this study, we found that administration of OVA/SND increased Th1-biased immune responses, including cytokine levels of IL-1α, IL-2, IL-12p40, IL-13, eotaxin, KC, MCP-1, TNF-α, and IFN-γ, compared with OVA/OPD (all *P*s *<*0.01), in addition to IL-1β, IL-3, IL12P70, MIP-1α, MIP-1β, RANTES, C-GSF, and GM-CSF (all *P*s *<*0.05), as shown in **Figure 5B**. Furthermore, OVA/SND stimulated a more robust increase in cytokine levels with respect to Th2 (IL-4, IL-5, IL-6, IL-9, IL-10) than did OVA/OPD (all *P*s *<*0.001; **Figure 5C**). In addition, OVA/SND stimulated an increase in cytokine levels with respect to inflammation (IL-1α, IL-1β, IL-2, IL-3, IL-12p40, IL-12P70, IL-13, IL-4, IL-5, IL-6, IL-9, IL-10, IL-17A; **Figure 5D**). Thus, OVA/SND could greatly activate and enhance cellular uptake (Th1/Th17) and, to some extent, induced humoral immune responses (Th2) after immunization.

### Preventative component protective effects against tumor

**Figure 6:**
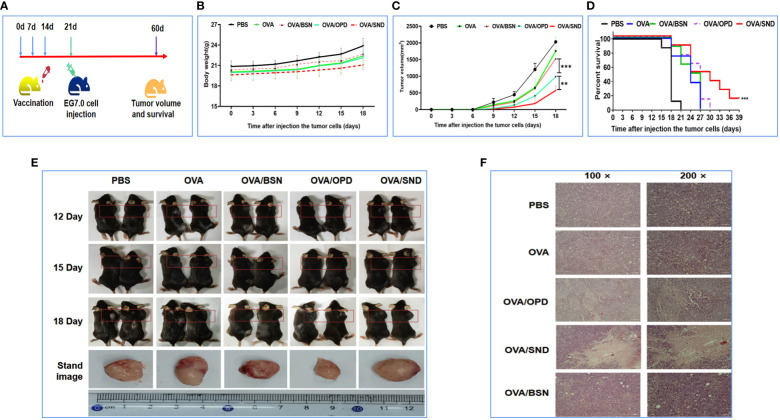
Preventative anti-tumor effects of the nanoadjuvant in combination with OVA. **(A)** Mouse vaccination procedure under the preventive model. **(B)** Body weight curves. **(C)** Average tumor growth curves under the preventive model. **(D)** Percent survival curves under the preventive model. **(E)** Representative photographs of tumor-bearing mice and tumors after different treatments, taken at 12, 15, and 18 days after treatment. **(F)** Assessment of tumor necrosis after staining with hematoxylin and eosin. For the survival analysis, differences were assessed by the log-rank test. **P < 0.01; ***P < 0.001.

OVA-expressing E.G7-OVA cells were used to evaluate the preventive component of the protective anti-tumor effects of OVA/SND in mice. Mice in the survival rate analysis group were inoculated subcutaneously in the right shoulder with 4 × 10^5^ E.G7-OVA cells 7 days after the final immunization, while mice in the tumor pathology analysis group were inoculated subcutaneously in the right shoulder with 1.5 × 10^5^ E.G7-OVA cells 7 days after the final immunization. The survival time of the mice and tumor volume after injection of tumor cells were monitored (**Figure 6A**). We found that there was no change in the weight of the mice (*P*>0.05, **Figure 6B**), but tumor growth was significantly inhibited in the OVA/SND group compared with the OVA-only group (*P*<0.01; **Figures 6C, E**). Tumor sections generally showed enlargement of tumor cells and nuclei. When cells undergo apoptosis, tumor tissues undergo cell contraction and chromosomal degradation. We found that, in the PBS-treated group, the tumors exhibited a fairly intact structure and regular shape. No necrotic areas were seen. However, a large number of infiltrating inflammatory cells were observed at ×100 and ×200 magnification in the OVA/SND group (**Figure 6F**). As can be seen from **Figure 6D**, 18 days after final immunization, tumor volume was significantly lower after treatment with OVA/SND than in the case of the other controls. In the prevention analysis, the SND adjuvant reduced tumor volume by 1/2 and prolonged median survival by 3 days, which confirms that stronger preventive effects occurred in the OVA/SND group than in the OVA/OPD and OVA groups.

### Therapeutic effects against tumor

**Figure 7:**
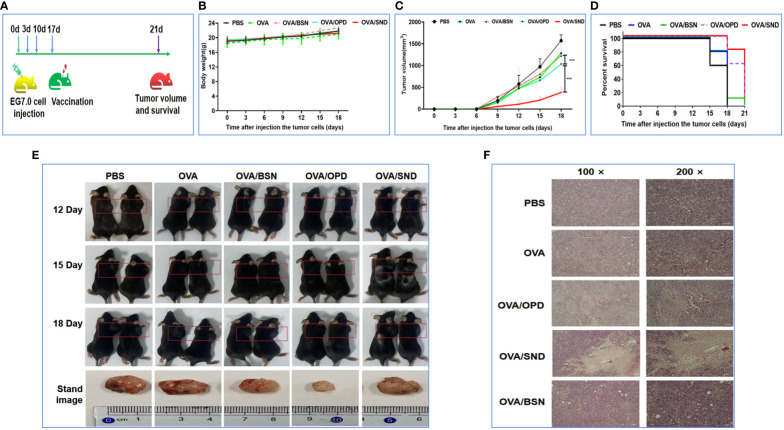
Therapeutic anti-tumor effects of the nanoadjuvant in combination with OVA. **(A)** Mouse vaccination procedure under the therapeutic model. **(B)** Body weight curves. **(C)** Average tumor growth curves under the therapeutic model. **(D)** Percent survival curves under the therapeutic model. **(E)** Representative photographs of tumor-bearing mice and tumors. **(F)** Assessment of tumor necrosis after staining with hematoxylin and eosin. For the survival analysis, differences were assessed by the log-rank test. ***P < 0.001.

To investigate the therapeutic effect, we first injected E.G7-OVA lymphocyte cells into the right shoulders of the mice on day 0 (4 × 10^5^ E.G7-OVA cells for the survival rate analysis group and 1.5 × 10^5^ E.G7-OVA cells for the tumor pathology analysis group). Mice were then immunized on days 3, 10, and 17 (**Figure 7A**). The body weight and tumor volume of the mice were recorded every three days; it was found that there was no change in their weight (*P*>0.05, **Figure 7B**), but tumor volume on day 18 was significantly lower in the OVA/SND group than in the OVA group (*P*<0.001, **Figures 7C, E**). We also found that, in the PBS-treated group, the tumors exhibited a fairly intact structure and regular shape. No necrotic areas were seen. However, a large number of infiltrating inflammatory cells were observed at ×100 and ×200 magnification in the OVA/SND group (**Figure 7F**). In addition, under the treatment model, the SND adjuvant reduced tumor volume by 2/3 and prolonged median survival by 3 days (**Figure 7D**). These results indicated that the vaccine exerted an important therapeutic effect on E.G7-OVA tumors.

### Antigen persistence of the nanoadjuvant in combination with Cy5.0-OVA

**Figure 8:**
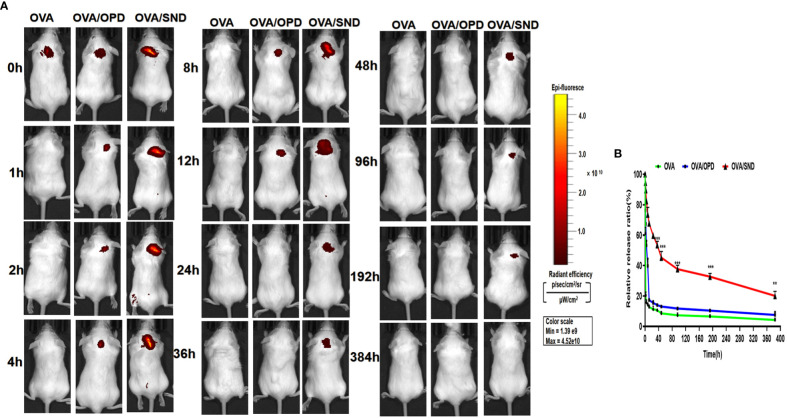
Antigen persistence at the injection site for the nanoadjuvant in combination with Cy5.0-OVA, measured using a Carestream FX PRO *in vivo* imaging system. **(A)** Representative fluorescence images and **(B)** quantitative fluorescence intensity indicating persistence of the antigen at the subcutaneous injection site. Data are expressed in the form mean ± SD (n = 3). **P < 0.01; ***P < 0.001.

To evaluate antigen persistence with the use of this nanoadjuvant in combination with Cy5.0-OVA, we prepared Cy5.0-OVA and employed IVIS to detect the amount of SND combined with Cy5.0-labeled OVA *in vivo*. Mice were injected with Cy5.0-OVA, Cy5.0-OVA/OPD, or Cy5.0-OVA/SND, and monitored at 0, 1, 2, 8, 12, 36, 48, 96, 192, and 384 h. The imaging and antigen release results are shown in **Figure 8**. We observed complete disappearance of fluorescence within 1 h and within 12 h after treatment with Cy5.0-OVA and Cy5.0-OVA/OPD, respectively. Meanwhile, complete disappearance occurred within 384 h after treatment with Cy5.0-OVA/SND, as shown in **Figure 8A**. Compared with Cy5.0-OVA/OPD and Cy5.0-OVA, fluorescence decreased slowly in the case of treatment with Cy5.0-OVA/SND; even 12 h after administration, it was stronger than that of the OVA antigens, as shown in **Figure 8B**. These results suggest that this system could delay the rapid release of OVA antigens and significantly prolong their period of residence *in vivo*.

## Discussion

Because tumor vaccines have the potential to induce effective anti-tumor immune responses, they have attracted widespread interest in tumor therapy (32). However, many challenges, including weak immunogenicity, off-target effects, and the immunosuppressive microenvironment hinder the broad clinical translation of these effects. To overcome these difficulties, efficient delivery systems for cancer vaccines have been designed (33). An excellent tumor vaccine should be able to trigger both cellular and humoral immunity, eliciting a robust and durable immune response (34). Aluminum adjuvants are well known to promote humoral immune responses to adsorb antigens and alter their immunological properties (35). Up to now, only a very small number of adjuvants have been approved as components of licensed human vaccines, but the development of new adjuvants that can safely enhance the adaptive immune response has been shown to be beneficial in vaccine-based efforts to target diseases that put billions of people at risk, including malaria, tuberculosis, infectious diseases, the human immunodeficiency virus (HIV), and COVID-19 (13). Therefore, the development of a new adjuvant for the development of tumor vaccines is of great significance.

Saponins are known to be triterpenoid glycosides isolated from natural sources such as the Quillaja saponins tree, and have been extensively studied as vaccine adjuvants. Advances in formulations making use of these led to the first licensed vaccines using saponin adjuvants, which used liposomal saponins and MPLA (Glaxo Smith Kline’s AS01 adjuvant for the Shingrix and Mosquirix vaccines). Despite their clinical importance, the methods by which saponins promote humoral and cellular immunity are still poorly understood (13).

In our previous report, we described the development of a powerful immunomodulator, OPD, which has been employed in a highly efficient and low-toxicity adjuvant system (NOD) constructed using nanoemulsion technology in dozens of natural products. These results showed that the adjuvant system exhibited strong adjuvant activity *in vivo* and *in vitro*, and the protection rate reached 100% (20). However, the question of whether this natural plant immunomodulator could induce a cellular immune response, especially in the form of a tumor vaccine, remained open. A self-nanoemulsion system can be defined as an isotropic mixture of oil, surfactant, and cosolvents, usually hydrophilic, which form a fine oil by introducing an aqueous emulsion in the aqueous phase (36). This new type of system for greatly improving drug dissolution has attracted attention within the field (37). Importantly, this form of vaccine nanoadjvuant has been found to elicit robust and complementary humoral and cellular responses characterized by the generation of a balanced Th1/Th2 immune response (38). Therefore, in this study, we selected and designed self-nanoemulsion system for use with OPD to improve the humor and cellular immune response to this tumor vaccine to a great extent.

Physical and chemical properties and adaptations, including shape, size, and charge, are essential in the development of this novel adjuvant (28). For example, TEM and SEM are high-precision instruments that have been widely used in the development of nanomaterials for many years in the scientific community. AFM had become promising technique for modeling of the nanomechanical roles of biological samples. Naturally, in addition to physical characteristics as determined by DSC, TG, FITR DLS, SDS-PAGE, and MTT, we examined drug interaction and particle size distributions; important characteristic changes in size, PDI, and zeta potential; protein damage; and cytotoxicity. These data (**Figures 1** and **2**) confirmed that the novel SND nanoadjuvant exhibited good properties and high stability.

The titers of specific IgG antibodies (IgG1 and IgG2c) were detected by ELISA. The titers of OVA-specific IgG, IgG1, IgG2a, IgG2b antibodies were significantly increased (5 weeks after immunization), indicating it progression toward a Th1-type immune response. The antibody levels in the OVA/SND group were significantly increased compared with those in the other (control) groups (**Figure 3**). IFN-γ released by effector T cells enhances the expression of immunosuppressive markers by tumor-associated lymphatic endothelial cells (39). At the same time, other studies have also shown that Th17 cells have a protective anti-tumor immune function. Overall, these data provide supporting evidence that IL-17 may play a dual role in human tumor immunity. Moreover, the activation of tumor-specific Th17 cells may promote the therapeutic efficacy of cancer vaccines (40). In our study, we found that CTL responses induced by the Th1/Th17 cellular response (**Figure 4**) were also dependent on IFN-γ, and type I IFN signaling by CD8^+^ T cell responses induced by OVA/SND were stronger for IFN-γ and IL-17 than those induced in the other groups. Furthermore, the detection of serum cytokines in immunized mice further demonstrated that OVA/SND predominantly elicited Th1/Th2/Th17-type immune responses after immunization (**Figure 5**); these results suggest that the protective effects of the novel nanoadjuvant in combination with OVA was mainly caused by specific humoral and cellular immune responses.

In our study, we also selected the OVA protein in combination with the novel nanoadjuvant for evaluation of anti-tumor efficacy. E.G7-OVA cells constitutively express OVA and have been used to study H-2Kb-restricted cytotoxic lymphocytes specific to OVA (41). Based on the results, this attack on E.G7-OVA cells was able to delay the normal growth of the tumor for a long time and with a high rate of success after vaccination. Adjuvant activity and the efficiency of delivery of the nano-vaccine might be the keys to enhancing the immune protective response *in vivo (* 38). The key to the anti-tumor effects in terms of prevention and treatment was the rapid induction of a cellular and humoral response after delayed antigen release (**Figure 8**). These data confirmed that this novel nano-adjuvant-encapsulated native plant immunostimulatory molecule is an excellent candidate for use as a tumor vaccine adjuvant that could reactivate the immune response and exert a potent suppressive effect on tumor growth. However, there are still various issues to be further investigated, such as molecular targets, multiple choice of tumor models, and so on.

## Conclusion

In the current study, we designed a novel anti-tumor nanoadjuvant (SND) vaccine based on OPD and modern self-nanoemulsifying technology. We found that this novel nanoadjuvant, which has good characteristics and high stability, could greatly improve the OVA antibody immune response (IgG, IgG1, IgG2a, and IgG2b); additionally, Th1/Th17 cellular immune levels of cytokines (IFN-γ, IL-1β, IL-4, and IL-17A) in splenocytes were both greatly enhanced at 0, 14, and 28 days after immunization by injection with the novel nanoadjuvant and antigen OVA. Importantly, this novel nanoadjuvant in combination with OVA was able to reinvigorate the Th1/Th2/Th17 immune response and enhanced both preventative and therapeutic anti-tumor effects. These results suggest that the SND is a promising and ideal adjuvant for use in cancer vaccines.

## Data availability statement

The original contributions presented in the study are included in the article/supplementary material. Further inquiries can be directed to the corresponding authors.

## Ethics statement

The animal experimental protocols were approved by the Laboratory Animal Welfare and Ethics Committee of the Third Military Medical University and performed according to the Guide for the Care and Use of Laboratory Animals (27).

## Author contributions

XL and ZS contributed equally to this work. YuY, HS and QZ designed the experiments and wrote the manuscript. XL, ZS, XZ, YaY, HLZ, DC, YT and QY carried out the experiments. HL, DL, YL and HZ analyzed the experimental results and revised the manuscript. All authors contributed to the article and approved the submitted version.
